# Oculopharyngodistal myopathy with coexisting histology of systemic neuronal intranuclear inclusion disease: Clinicopathologic features of an autopsied patient harboring CGG repeat expansions in *LRP12*

**DOI:** 10.1186/s40478-020-00945-2

**Published:** 2020-06-03

**Authors:** Rie Saito, Hiroshi Shimizu, Takeshi Miura, Norikazu Hara, Naomi Mezaki, Yo Higuchi, Akinori Miyashita, Izumi Kawachi, Kazuhiro Sanpei, Yoshiaki Honma, Osamu Onodera, Takeshi Ikeuchi, Akiyoshi Kakita

**Affiliations:** 1 Department of Pathology, Brain Research Institute, Niigata University, 1-757 Asahimachi, Chuo-ku, Niigata, 951-8585 Japan; 2 Department of Neurology, Sado General Hospital, Niigata, Japan; 3 Department of Neurology, Brain Research Institute, Niigata University, Niigata, Japan; 4 Department of Molecular Genetics, Brain Research Institute, Niigata University, Niigata, Japan; 5 Comprehensive Medical Education Center, Niigata University School of Medicine, Niigata, Japan

**Keywords:** Oculopharyngodistal myopathy, Neuronal intranuclear inclusion disease, Oculopharyngeal myopathy with leukoencephalopathy, Noncoding CGG expansions, Neuropathology

Unstable tandem repeat expansions are important genetic motifs that underlie various neurological disorders. Two interesting aspects of noncoding CGG expansions have recently attracted attention: pleiotropy in which expanded CGG repeats in *NBPF19* cause two disorders that were formerly unlinked, namely neuronal intranuclear inclusion disease (NIID) [2, 4] and essential tremor [6], and the clinical spectrum resulting from similar CGG repeat expansion motifs in different genes, namely *NBPF19*, *LRP12*, and *LOC642361*, which cause NIID, oculopharyngodistal myopathy (OPDM), and a new overlapping disease oculopharyngeal myopathy with leukoencephalopathy (OPML), each designated as NIID1, OPDM1, and OPML1, respectively [2]. Despite such improved understanding of their clinical/genetic aspects, the pathological features of these disorders remain elusive.

Recently, we have experienced a two-generation pedigree of clinically typical OPDM [1] that affected the father and one of his two children (Fig. 1a); the latter (the proband) was autopsied and shown to harbor CGG repeat expansions in *LRP12* (Fig. 1b), establishing the diagnosis of OPDM1. Both the proband and his father developed adult-onset ptosis, distal-dominant slowly progressive myopathy with rimmed vacuoles (RVs) (Fig. 2a-d) [3], dysphagia, and external ophthalmoplegia (Additional file 1). Ultrastructural analysis of the necropsied scalenus muscle revealed tubulofilamentous inclusions in the muscle fiber nuclei [3]; occasionally, two inclusions were observed in one nucleus (Fig. 2e, f). Importantly, these inclusions were undetectable by routine histology or ubiquitin immunohistochemistry. In addition to the muscle pathology, a general autopsy of the proband unexpectedly revealed coexisting NIID histopathology. Almost all organs including the central and peripheral nervous systems, except for the skeletal muscles, showed abundant round, eosinophilic intranuclear inclusions (Fig. 3a) that were positive for ubiquitin (Fig. 3b) and p62 and most frequently detectable in the sympathetic (Fig. 3c) and dorsal root ganglia. In these two regions, 54 and 41% of neurons possessed p62-positive nuclear inclusions, and 3 and 5% possessed two inclusions, respectively. In the brain, these inclusions were observed in neurons (Fig. 3d), astrocytes (Fig. 3e), and Schwann cells. The distribution (Table 1) and ultrastructural features (Fig. 3f, g) of the inclusions were indistinguishable from those in NIID1 [4, 5].

**Fig. 1:**
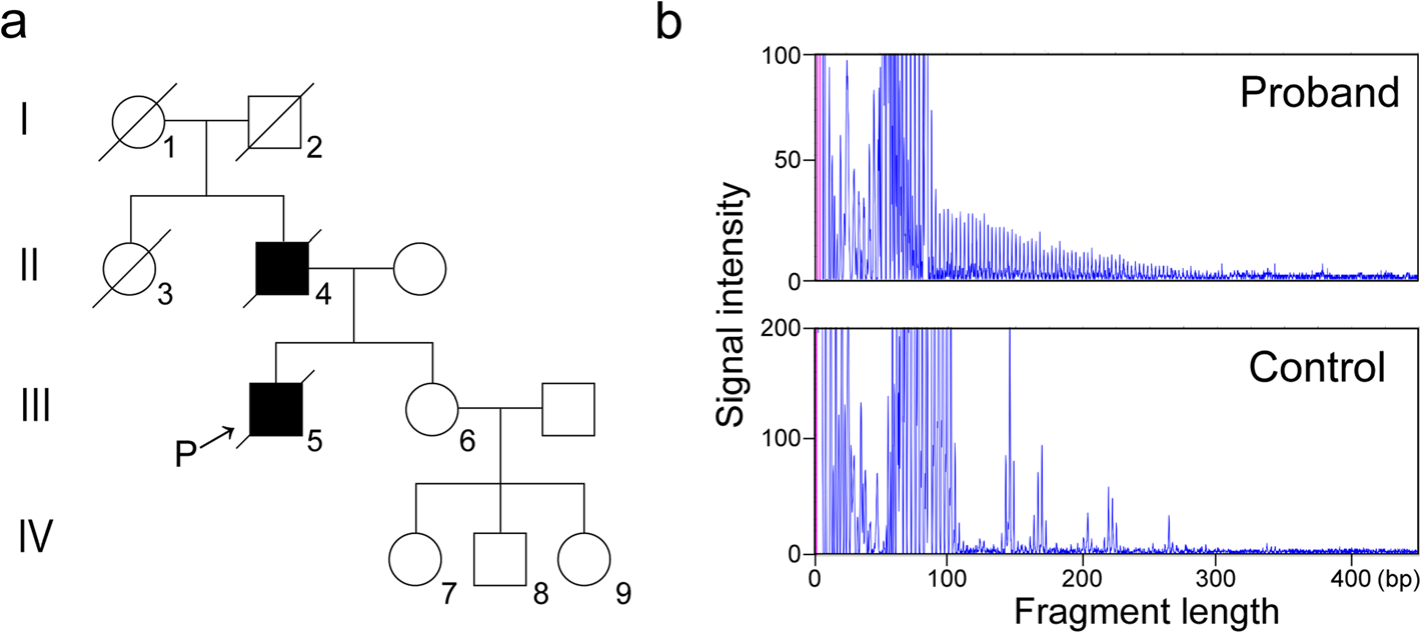
Detection of CGG repeat expansions in *LRP12.***a** Pedigree of the family. The individuals suffering from OPDM are indicated by solid symbols. The family shows autosomal dominant inheritance. **b** Repeat-primed PCR analysis using frozen frontal cortex obtained from the proband (**a**, III-5) demonstrates CGG repeat expansions in *LRP12* (*upper panel*), whereas no such expansions are evident in the control (*lower panel*). In the proband, there were no pathological tandem repeat expansions in *FMR1*, *NBPF19*, *LOC642361/NUTM2B-AS1*, or *PABPN1*, genetically excluding the possibility of fragile X tremor/ataxia syndrome (FXTAS), neuronal intranuclear inclusion disease 1 (NIID1), oculopharyngeal myopathy with leukoencephalopathy 1 (OPML1), or oculopharyngeal muscular dystrophy (OPMD). P, proband

**Fig. 2:**
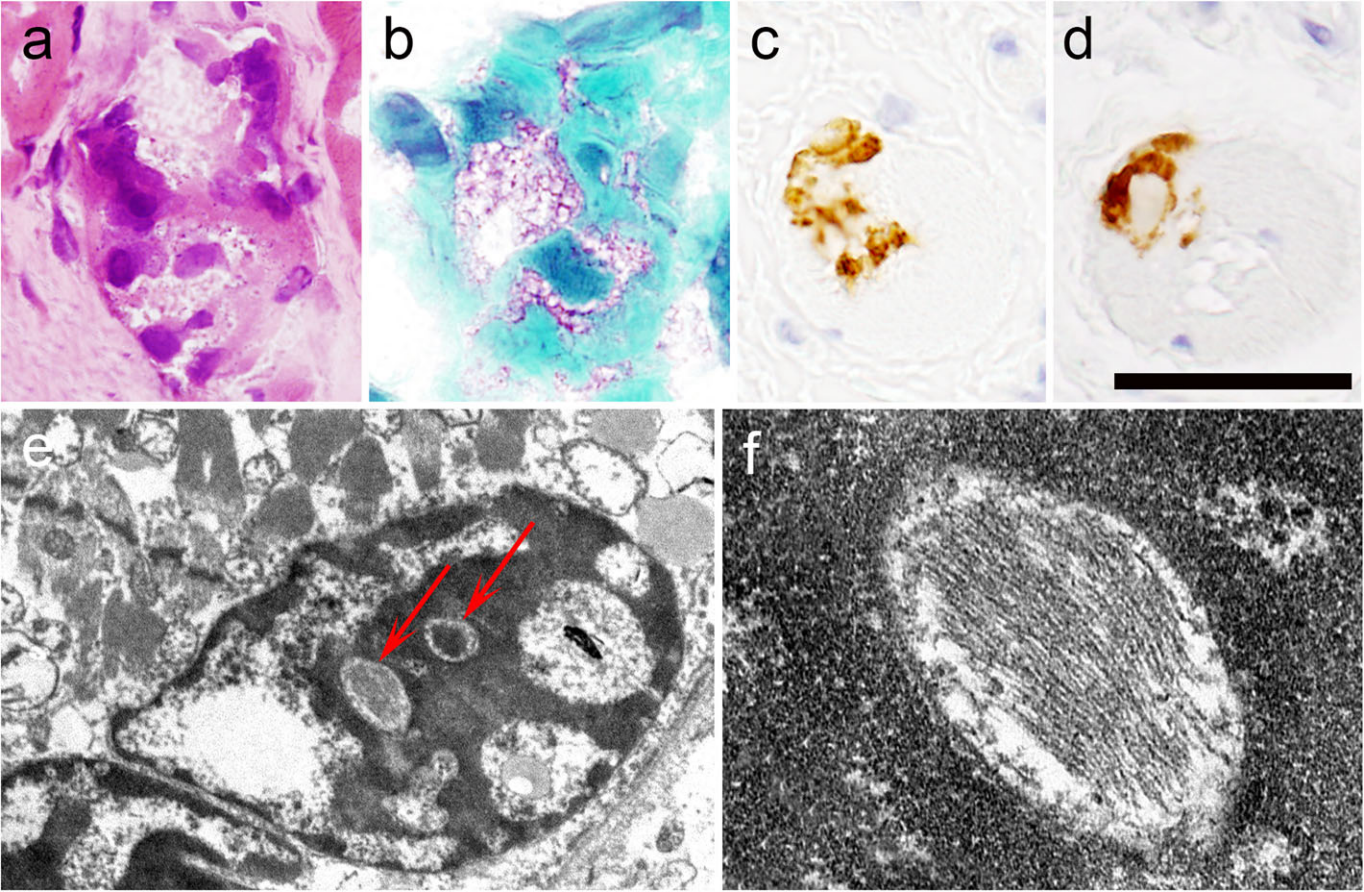
Pathology of the skeletal muscles. **a**-**d** Rimmed vacuoles (RVs). Skeletal muscles show typical RVs on staining with HE (**a**) and modified Gomori trichrome (**b**). RVs are positive for p62 (**c**) and phosphorylated TDP-43 (**d**). **e**, **f** Intranuclear inclusions of the skeletal muscles. These inclusions were detectable only by electron microscopy (**e**). They had no obvious limiting membranes and were composed of straight filaments about 13–18 nm in diameter (**f**). **a**, **b** anterior tibial muscle, biopsy; **c**, **d** striated muscle of the esophagus, necropsy; **e**, **f** scalenus muscle, necropsy. Scale bar: **a**-**d** = 40 μm, **e** = 2 μm, **f** = 500 nm.

**Fig. 3:**
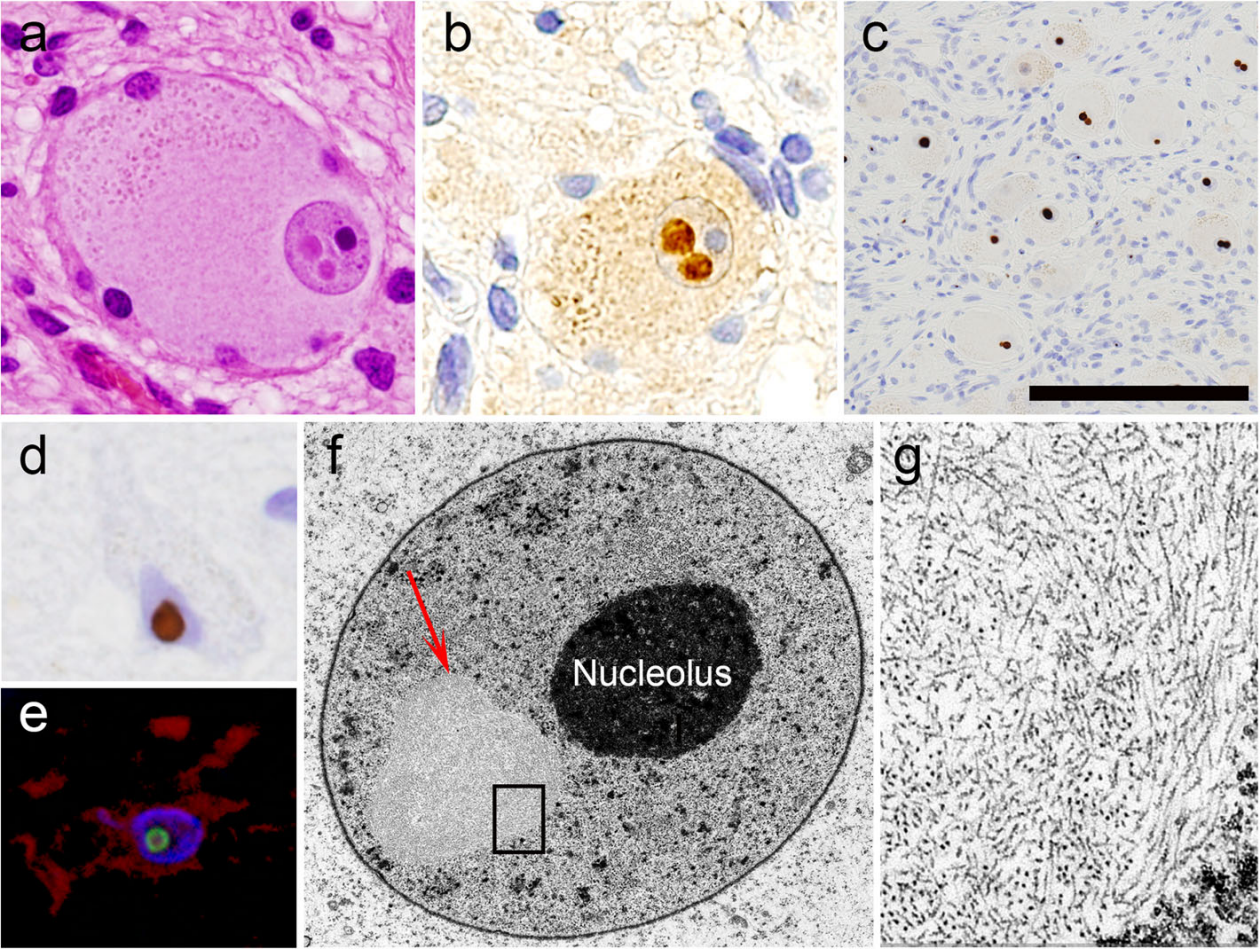
Intranuclear inclusions in extra-skeletal muscle organs. **a**-**c** Sympathetic ganglia. Two eosinophilic neuronal intranuclear inclusions with surrounding halos are evident on HE staining (**a**). The inclusions are positive for ubiquitin (**b**). Numerous inclusions are revealed by p62-immunohistochemistry (**c**). **d** A p62-positive neuronal intranuclear inclusion in the transentorhinal cortex. **e** A p62-positive intranuclear inclusion (green) in a GFAP-positive astrocyte (red) in the temporal cortex. Double-labeling immunofluorescence. **f**, **g** Ultrastructure of a sympathetic ganglion neuron containing an intranuclear inclusion (**f**, *arrow*). The inclusion is composed of fine filamentous structures without limiting membranes. Scale bar: **a**, **b** = 40 μm; **c** = 150 μm; **d**, **e** = 25 μm; **f** = 5 μm; **g** = 750 nm

**Table 1:** Distribution and density of p62-positive intranuclear inclusions in the proband

**CNS**	Neuron	Astrocyte	**PNS**	Neuron	Schwann cell	
***Cerebrum***			Sympathetic ggl	3	2	
Frontal^a^	1/n.a.	1/1	Dorsal root ggl	3	2	
Motor^a^	2/n.a.	1/1	Myenteric ggl	2	2	
Temporal^a^	2/n.a.	2/1				
Parietal^a^	1/n.a.	1/1	**Visceral organs**			
Occipital^a^	2/n.a.	1/1	Kidney	Renal tubule	Glomerulus	
Hippocampus^b^	2/3/1	1/1/1		2	2	
Amygdala	2	1	Liver	Hepatocyte		
NBM	1	1		0		
Caudate/putamen	1/1	1/1	Pancreas	Acinar cell	Ductal cell	
Globus pallidus i/e	1/1	1/1		1	1	
Thalamus	2	1	Adrenal	Cortex	Medulla	
***Brainstem***				1	1	
Midbrain tectum	2	1	Heart	Muscle cell		
Oculomotor nuc	2	2		1		
Red nuc	2	2	Skeletal muscle	Muscle cell		
Substantia nigra	3	2		0		
Locus ceruleus	2	1				
Facial nuc	2	1	**Somatic cells**	Adipocyte	EC	SMC
Pontine nuc	1	1		1	1	2
Hypoglossal nuc	2	0				
IO	2	1				
***Cerebellum***^c^	1/n.a./0	1/1/1				
***Spinal cord***						
Anterior horn	2	1				
IML	2	0				
Clarke’s column	0	0				
Posterior horn	2	1				
Wm	0	1				
***Others***	Ependymal cell	Choroid plexus				
	0	2				

The density of inclusions was graded according to the percentage of inclusion-bearing cells: 0, none; 1, 0–10%; 2, 10–40%; 3, > 40%

^a^ cortex/white matter; ^b^ CA1/CA4/dentate gyrus; ^c^ Cerebellar cortex/white matter/dentate nucleus; wm, white matter; n.a., not available; NBM, nucleus basalis of Meynert; i/e, internal segment/external segment; nuc, nucleus; IO, inferior olive; IML, intermediolateral cell column; ggl, ganglion; EC, endothelial cell; SMC, smooth muscle cell

These findings suggest that the disease process of OPDM1 is not limited to the oculopharyngeal muscles and can affect various extra-skeletal muscle organs including the central and peripheral nervous systems. Furthermore, the observed ubiquitinated inclusions were indistinguishable from those in NIID1, strongly supporting the hypothesis that such transcribed expanded CGG repeats are commonly involved in the development of OPDM-NIID spectrum disorders, irrespective of the genes where the repeats are located [2].

A major question that naturally arises is whether or not the NIID1-like lesions with intranuclear inclusions described here caused symptoms. The major clinical features of NIID such as dementia, parkinsonism, or neurogenic muscular weakness were not observed in the present pedigree, implying that in OPDM-NIID spectrum disorders, development of clinical symptoms is not merely dependent on the transcribed CGG repeats, but also associated with dysfunction of the mutated genes. Unfortunately, due to agonal brain edema and absence of brain MRI data, we could not determine the presence or absence of cerebral white matter degeneration. In OPDM1 and OPML1, however, lesions in the autonomic nervous system may commonly develop. Three out of six patients of the OPML1 pedigree had gastrointestinal symptoms [2], while the present OPDM1 patient showed the highest density of intranuclear inclusions in autonomic ganglia neurons, although the symptoms were unremarkable. Currently, it remains unclear whether the hypertrophic cardiomyopathy observed in the present OPDM1 patient (Additional file 1), or the rare occurrence of dilated cardiomyopathy in patients with OPDM [7] and OPML1 [2] is also associated with CGG repeat expansions. Further studies are necessary to clarify any extra-muscular organ involvement and associated symptoms that might have been masked by more obvious features relevant to muscle pathology.

Noncoding repeat expansions cause diseases through several mechanisms such as loss of expression of the repeat-containing genes and RNA gain-of-function, the latter including 1) repeat associated non-AUG (RAN) translation that generates toxic, often ubiquitinated aggregation-prone proteins and 2) formation of RNA foci that sequester RNA-binding proteins. In the present autopsied patient with OPDM1, the inclusions in the skeletal muscles were not ubiquitinated and visible only by electron microscopy, whereas those in extra-skeletal muscle organs were ubiquitinated and detectable by routine histology. The mutually exclusive distribution and different natures of these two types of inclusion may reflect a difference in the pathomechanisms whereby they are recruited to skeletal muscles or extra-skeletal muscle organs. Interestingly, like OPDM1, no eosinophilic, ubiquitinated inclusions have been reported in skeletal muscles in OPML1 or NIID1 (Table 2). It would be of great interest if extensive electron microscopy examination revealed that inclusions in skeletal muscles morphologically resembled those observed in OPDM1.

**Table 2:** Intranuclear inclusions in diseases associated with noncoding CGG repeat expansions

	NIID1 [4, 5] *NBPF19*	OPML1 [2] *LOC642361/* *NUTM2B-AS1*	OPDM1 Present study *LRP12*
Skeletal muscles			
HE/ubiquitin	Undetectable	n.a.	Undetectable
Electron-microscopy	n.a.	n.a.	Tubulofilamentous
Extra-skeletal muscle organs			
HE/ubiquitin	Widespread	n.a.	Widespread
Electron-microscopy	Fine filamentous	n.a.	Fine filamentous

*OPDM* oculopharyngodistal myopathy, *OPML* oculopharyngeal myopathy with leukoencephalopathy, *NIID* neuronal intranuclear inclusion disease, *n.a.* not available

In conclusion, we have presented an OPDM1 pedigree associated with *LRP12* including the first reported genetically confirmed autopsy case showing histological evidence of systemic NIID-1 like lesions. Further clinicopathological and molecular studies will help to clarify how noncoding CGG expansions in different genes form the NIID-OPDM spectrum, and the pathomechanism underlying the similarities and differences among these disorders.

## Supplementary information

**Additional file 1.**

## Data Availability

The datasets used and analysed during the current study available from the corresponding author on reasonable request.

## Abbreviations

OPDM: Oculopharyngodistal myopathy
NIID: Neuronal intranuclear inclusion disease
OPML: Oculopharyngeal myopathy with leukoencephalopathy
RV: Rimmed vacuole
