# Supplementary material for: Oculopharyngodistal myopathy with coexisting histology of systemic neuronal intranuclear inclusion disease: Clinicopathologic features of an autopsied patient harboring CGG repeat expansions in LRP12
Source: Acta Neuropathol Commun. 2020 Jun 3;8:75. doi: 10.1186/s40478-020-00945-2 (PMC7268649; doi:10.1186/s40478-020-00945-2)
Supplement: Supplementary file 1 — Additional file 1. [file 40478_2020_945_MOESM1_ESM.pdf]

Additional file 1

Acta Neuropathologica Communications

Letter to the editor

Oculopharyngodistal myopathy with coexisting histology of systemic neuronal intranuclear inclusion disease: Clinicopathologic features of an autopsied patient harboring CGG repeat expansions in *LRP12*

Rie Saito[1], Hiroshi Shimizu[1], Takeshi Miura[2,3], Norikazu Hara[4], Naomi Mezaki[2,3], Yo Higuchi[3,4], Akinori Miyashita[4], Izumi Kawachi[3,5], Kazuhiro Sanpei[2], Yoshiaki Honma[2], Osamu Onodera[3], Takeshi Ikeuchi[4], Akiyoshi Kakita[1]

Departments of Pathology[1], Neurology[3], Molecular Genetics[4], Brain Research Institute, Niigata University, Niigata, Japan.

[2] Department of Neurology, Sado General Hospital, Niigata, Japan.

[5] Comprehensive Medical Education Center, Niigata University School of Medicine, Niigata, Japan.

Correspondence to: Hiroshi Shimizu, MD, PhD

Department of Pathology, Brain Research Institute, Niigata University, 1-757 Asahimachi, Chuo-ku, Niigata 951-8585, Japan.

E-mail: hshimizu@bri.niigata-u.ac.jp

Clinical presentation

Methods

References

Clinical presentation

The proband (Fig. 1a: III-5) initially noticed bilateral ptosis at the age of 36 years. Two years later, he developed muscle cramps in his left foot and muscle weakness in his hands. On neurological examination, he had a myopathic face, external ophthalmoplegia, dysphonia (nasal voice), and dysphasia. Atrophy and weakness of the distal muscles were evident, while the proximal muscles were well preserved. The serum creatine kinase (CK) level was elevated (821 U/L). Electromyography demonstrated myopathic changes in the first dorsal interosseous muscles of the hand and anterior tibialis. The results of a nerve conduction study were normal. Muscle biopsy revealed myopathic change with rimmed vacuoles. As *PABPN1* showed a normal number of CGG repeats, oculopharyngeal muscular dystrophy (OPMD) was excluded. The clinical course and results of muscle biopsy were closely similar between the proband and his father (Fig. 1a: II-4). Therefore, the affected members of the pedigree were diagnosed as having autosomal-dominant oculopharyngodistal myopathy (OPDM). By the age of 48 years, the proband had become wheelchair dependent. Due to the progression of dysphasia and respiratory distress, gastrostomy and tracheostomy with mechanical ventilation were initiated at the age of 57 years. He died of liver cirrhosis aged 64 years. Neither the proband nor his father had any history of cognitive decline or neuropathy. A general autopsy of the proband revealed hypertrophic cardiomyopathy and liver cirrhosis that had progressed from non-alcoholic fatty liver disease (NAFLD). The brain showed no pathological features suggestive of complications arising from Alzheimer's disease (ABC score: A2B0C3 [3]) or Parkinson's disease (Lewy body disease: none [2]).

Methods

Neuropathology

The brain, spinal cord, skeletal muscles (iliopsoas, scalenus, and thyroarytenoid muscles, diaphragm, and tongue), and general visceral organs of the proband obtained at autopsy were fixed with 20% buffered formalin, and multiple tissue blocks were embedded in paraffin. Histological examination was performed on 4-μm-thick sections stained with hematoxylin and eosin and by the Klüver-Barrera method. In addition, selected sections were immunostained with antibodies against ubiquitin (Dako, Glostrup, Denmark; 1:800), p62 (BD Transduction Laboratories, CA, USA; 1:1000), and phosphorylated TDP43 (Cosmo Bio, Tokyo, Japan; 1:5000). Antibodies against amyloid β 11-28 (IBL, Gunma, Japan; 1:50), phosphorylated tau (Fujirebio, Gent, Belgium; 1:200) and phosphorylated α-synuclein (Wako, Saitama, Japan; 1:1000) were used to assess senile pathologic changes based on "ABC" score [3] and the fourth consensus report of the DLB consortium [2]. Bound antibodies were visualized by the peroxidase-polymer-based method using a Histofine Simple Stain MAX-PO kit (Nichirei, Tokyo, Japan) with diaminobenzidine as the chromogen. Immunostained sections were counterstained with hematoxylin.

A double-labeling immunofluorescence study was performed on sections obtained from the temporal cortex and white matter using mouse monoclonal anti-p62 (BD Transduction Laboratories, CA, USA; 1:500) with rabbit polyclonal anti-glial fibrillary acidic protein (Dako, Glostrup, Denmark; 1:400). The second antibodies used were Alexa Fluor 488 goat anti-mouse IgG (Molecular Probes Eugene, OR, USA; 1:1000) and Alexa Fluor 555 goat anti-rabbit IgG (Molecular Probes Eugene, OR, USA; 1:1000). The sections were treated with Autofluorescence Eliminator Reagent (Millipore, MA, USA), mounted under glass coverslips using VectaShield mounting medium with nuclear stain (Vector Laboratories, CA, USA) and analyzed using a Carl Zeiss confocal laser scanning microscope (LSM510-V4.0).

For electron microscopy, formalin-fixed scalenus muscle and sympathetic ganglia of the proband were post-fixed with 1% osmium tetroxide, dehydrated through a graded ethanol series, and embedded in Epon 812. Ultrathin sections were then cut and stained with uranyl acetate and lead citrate. Ultrathin sections were cut and examined with a Hitachi H-7100 electron microscope at 75 kV.

Frozen samples of the biopsied anterior tibial muscle of the proband were processed for routine histochemistry, as well as for immunohistochemistry using antibodies against ubiquitin and p62.

Genetic analysis

According to Ishiura *et al.* [1], we performed repeat-primed PCR in a reaction volume of 10 μl. Subsequent fragment analysis was conducted on an Applied Biosystems 3130xl Genetic Analyzer (Thermo Fisher Scientific, Waltham, MA, USA). The data were analyzed using GeneMapper version 4.1 (Thermo Fisher Scientific) to visualize signals showing repeat length.

References

1. Ishiura H, Shibata S, Yoshimura J, Suzuki Y, Qu W, Doi K et al (2019) Noncoding CGG repeat expansions in neuronal intranuclear inclusion disease, oculopharyngodistal myopathy and an overlapping disease. Nat Genet. 51:1222-1232.
2. McKeith IG, Boeve BF, Dickson DW, Halliday G, Taylor JP, Weintraub D et al (2017) Diagnosis and management of dementia with Lewy bodies: fourth consensus report of the DLB Consortium. Neurology 89:88-100.
3. Montine TJ, Phelps CH, Beach TG, Bigio EH, Cairns NJ, Dickson DW et al (2012) National institute on Aging-Alzheimer's association guidelines for the neuropathologic assessment of Alzheimer's disease: a practical approach. Acta Neuropathol. 123:1-11.
